# Broad-class volatile organic compounds (VOCs) detection via polyaniline/zinc oxide (PANI/ZnO) composite materials as gas sensor application

**DOI:** 10.1016/j.heliyon.2023.e13544

**Published:** 2023-02-05

**Authors:** Juanito Raphael F. Foronda, Lugas Gada Aryaswara, Gil Nonato C. Santos, Swathi N.V. Raghu, Muhammad Akhsin Muflikhun

**Affiliations:** a Physics Department, De La Salle University, Manila, Philippines; b Mechanical and Industrial Engineering Department, Gadjah Mada University, Indonesia; c Chemistry and Structure of Novel Materials (CSnM), University of Siegen, Germany; d Center for Advanced Manufacturing, Structural Engineering (CAMSE), Gadjah Mada University, Indonesia

**Keywords:** Chemical oxidative process, Selectivity, Gas sensing measurement, Pattern-recognition, Drop cast method

## Abstract

Metal-oxide doped conductive polymers have been investigated as sensors in the field of gas-sensing. Recent developments have highlighted the role of intrinsically conductive polymers, that have reportedly offered high surface response towards the detection of volatile organic compounds (VOCs). In this work, we optimize the development of gas-sensors made of Polyaniline/Zinc oxide (PANI/ZnO) composite, capable of detecting a varied class of VOCs such as, ammonia, acetone, formaldehyde, methanol, and ethanol. The conductivity of these sensors is evaluated at room temperature and are investigated until saturation. In addition to the final application, this work also focusses on the synthesis strategies to achieve an ‘optimal’ matrix-to-additive ratio, such that superior chemical response is paralleled with mechanical robustness for PANI based sensors. The PANI/ZnO composites are casted into sensors bearing different additive ratios, via a drop-casting method and the same is evaluated for its formability and mechanical behavior. Physio-chemical characterization was performed using Fourier Transform Infrared Spectroscopy (FTIR), Scanning Electron Microscope (SEM), and Energy Dispersive X-ray Analysis (EDX) and we report on an exceptional selectivity for ammonia with an average sensor response of 3496.67 mV by all the sensors, when fabricated using different matrix-additive ratios. This result is superior to what is observed for Pure- PANI sensors that were selective only to methanol and ethanol. The addition of ZnO in the smallest fraction, already offers a broader range of selectivity, e.g., PANI/ZnO 90:10 sensor was selective to formaldehyde as assessed using pattern recognition.

## Abbreviations

VOCs: Volatile organic compounds
PANI: Polyaniline
ZnO: Zinc oxide
FTIR: Fourier Transform Infrared Spectroscopy
SEM: Scanning Electron Microscope
EDX: Energy Dispersive X-ray Analysis
AC: Alternating current
DC: Direct current

## 1 Introduction

The rapid growth of industrialization has emerged phenomenal technological advances alongside several adverse environmental effects. Volatile organic compounds (VOCs), such as toxic gases and pollutants that result from industrial activities, are dangerous for human cardiac and respiratory systems [[1], [2], [3]]. Challenges pertaining to the detection, monitoring, and analysis of VOCs is a subject of increasing interest in the field of gas sensor development. Even though numerous gas sensor devices are available commercially, there exist gaps in the enhanced reliability and sensitivity of devices for accurate environmental monitoring [[4], [5], [6]]. Several studies continue to explore new material compositions to augment gas-sensing material properties. Conductive polymers have been studied because of their wide applications owing to the π-electron configuration within the polymeric structure [7]. The previous works showed that conducting polymers have several advantages, such as ease of synthesis, room temperature operation, short response time, and high sensitivity to various gases, which make conducting polymers a promising material for gas sensing [[8], [9], [10], [11]].

Many kinds of conducting polymers have been studied as gas-sensing materials and multifunctional materials such as polystyrene sulfonate (PEDOT:PSS), polypropylene, polypyrrole, polyacetylene, polythiophene, and polyaniline [[12], [13], [14], [15]]. PANI is one of the most superior materials for the gas sensing device since it outperforms polypyrrole as a result of enhanced sensitivity and shorter response time [16,17]. The ease of polymerization, abundant availability, lower cost, optical properties, photoelectrical properties, high sensitivity, and tunable electrical conductivity are several excellent properties inherent to PANI [18,19]. The tunable conductivity in PANI is caused by transferring and carrying load throughout the length of the polymer backbone and placing its hop in the polymer chain. Subsequently, the polyclonal molecule reacts with a deprotonation or protonation agent, which easily promotes a redox reaction at room temperature [[20], [21], [22], [23]]. However, PANI lacks mechanical strength and chemical stability [24]. This limitation is overcome by doping with other organic or inorganic materials and can consequentially enhance PANI's performance. Many studies about polymer blends, pure polymer film, and polymer-inorganic composites have been carried out. Several results have reported polymer-inorganic composite as the best solution to improve mechanical strength, gas sensing performance, optical properties, and electrical conductivity due to increased active sites as a result of doping [[25], [26], [27]]. Recently, different gas sensing materials such as poly (diphenylamine)/zinc oxide nanocomposites also being evaluated by researchers with promising results [28].

It has been reported that combining inorganic and organic materials in the form of composite or nanocomposite can enhance performance and generate unique characteristics for gas sensing devices [23]. In light of this, metal oxides are favorable candidates due to their intrinsic electronic properties, in addition to also being used in the field of anti-bacterial, structural, and electrical applications [[29], [30], [31], [32]]. ZnO is an inorganic metal oxide that has specific characteristics, such as: wide bandgap, excellent mechanical strength, good chemical stability, nontoxicity, high conductivity, large exciton binding energy, and in powdered form has a high surface area which favors increased throughput that demonstrates good applicability in gas sensing devices [[33], [34], [35], [36]]. Despite its advantages, ZnO is not as efficient in operating at room temperature. The high-temperature operation can reduce the gas-sensing device's lifetime and increase the power consumption cost [37]. Previous studies showed that combining ZnO and PANI can be applied in different applications. ZnO is an n-type semiconductor, and PANI is a p-type semiconductor. A *P*–N heterojunction can be formed upon the combination, leading to a potentially favorable gas-sensing performance [[38], [39], [40]].

The present manuscript thoroughly investigates, optimal material composition and its influence on gas sensing behavior at room temperature. In addition to extensive evaluation of the fabricated sensor's ability to detect various classes of VOCs, this manuscript also evaluates the physio-chemical and mechanical characteristics of the developed composite. This to our knowledge, remains one of the few works that documents an exhaustive list of properties in a singly reported-work, namely focusing on the synthesis and characterization of PANI and PANI/ZnO composite as a gas sensor. These were then evaluated for different gas combinations such as, Ammonia, acetone, formaldehyde, methanol, and alcohol. The improvements in mechanical robustness of the developed sensors were investigated as a function of reinforcing particles, I.e., ZnO weight percentage (wt.%) loading ratios on the PANI matrix as compared to pure PANI. PANI and PANI/ZnO composite were synthesized via the in-situ chemical oxidative polymerization method and were extensively characterized using SEM, EDX, FTIR, complimented by mechanical testing via atwo-point probe technique.

## 2 Materials and methods

### 2.1 Materials and synthesis of the PANI and PANI/ZnO composite

**Fig. 1:**
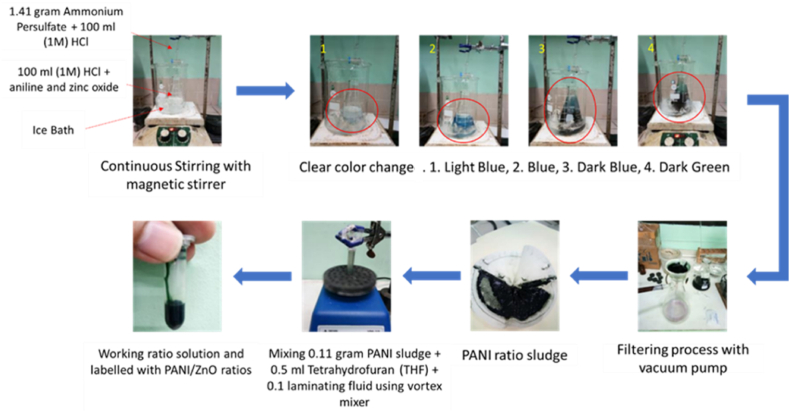
Experimental flowchart of PANI and PANI/ZnO Composite Synthesis.

In this study, several materials were used were supplied from different sources, as listed follow: Aniline monomer (Uni-Chem Chemical Reagents), Ammonia solution 30% and ammonium persulfate (Loba Cheme), ZnO (Sigma Aldrich), Hydrochloric acid 37% (RCI LabscanLtd.), Methanol and acetone (Mega Source Scientific Supplies), Absolute ethanol, and formaldehyde (JT Baker and Univar). The Chemical oxidative polymerization method was used to synthesize PANI and PANI/ZnO composite [41,42]. The modified chemical oxidative polymerization process is schematically detailed as shown in Fig. 1. Six different weight percentage ratios (% wt.) were evaluated in this research, and the values of aniline and zinc oxide. The detailed methods and the composition of samples are described in Appendix A1.

### 2.2 Polyaniline (PANI) and polyaniline/zinc oxide (PANI/ZnO) characterization

Phenom XL Desktop Scanning electron Microscope (SEM) and Energy Dispersive X-ray Spectroscopy (EDX) were used to investigate surface morphologies and chemical compositions of PANI and PANI/ZnO in a detailed scale. Fourier Transform Infrared Spectroscopy (FTIR) from Agilent Cary 630 Spectrometer was conducted to analyze the presence of surface functional groups. Resistance of all samples were carried out using a simple fluke multimeter. Keithley source measure unit 2450 two-point probe was used to obtain I–V characteristic scans. The gas sensing measurements were conducted using Sparkvue with voltage as the output signal. The parameters measured were sensor response, response time, recovery time, repeatability, and the selectivity of analytes. Analytes used were methanol, ethanol, ammonia, acetone, and formaldehyde.

### 2.3 Sensor fabrication and gas sensing set-up

**Fig. 2:**
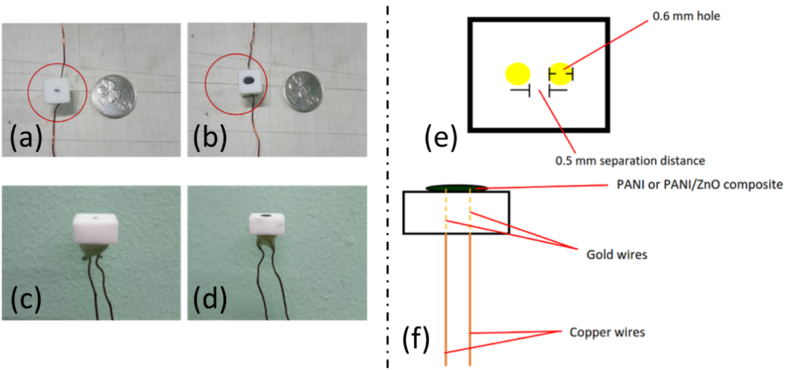
Actual and schematic of the gas sensor, where a) top view of substrate next to a coin (d 20 mm) for size reference; b) top view of sensor; c) side view of the substrate; d) side view of the sensor; e) Teflon substrate with dimensions; f) side view of the sensor.

The sensors were fabricated using the drop cast method. Teflon was used as a substrate for the sensing material with dimensions 10 mm × 10 mm x 5 mm. The Teflon substrate was designed such that, at the central cross-section has two 0.6 mm diameter holes that are 0.5 mm apart in addition to two gold wires soldered with copper, paired through the holes. These wires acted as the electrode. Drop cast was conducted for each ratio-variation of polyaniline, dropped over the substrate, and dried in room temperature (25 °C) for 6–12 h. The actual and graphical gas sensor are schematically shown in Fig. 2. It is shown in Fig. 2(a–d), the size of the gas sensor are quite small and compact, while the Teflon substrate with dimensions and another side view of the sensor are shown in Fig. 2(e–f).

**Fig. 3:**
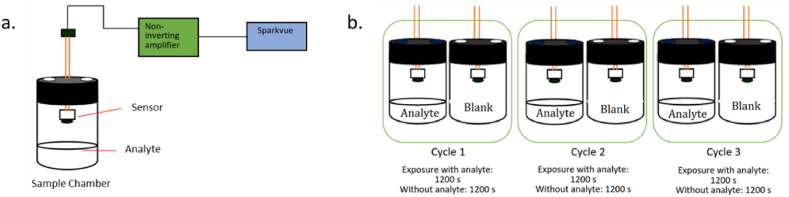
Diagram of a) gas sensing set-up schematically; b) cycle run time.

In the present study, a static headspace analysis was incorporated with the schematic diagram of the gas sensing set-up, as seen in Fig. 3. The sensor was reversed inside the sample chamber from a 60 mL amber bottle with holes on the cap (Fig. 3 (a)). The sensor becomes a chemistor to change the signal when exposed to the analyte. A Wheatstone bridge differential amplifier was used as an unknown resistance finder in the resistive bridge network by comparing the input voltage across the resistor. The sparkvue was used as the output reader for voltage as a function of time. In the test, there are three 40-min cycles (Fig. 3 (b)). The cycles were divided into 20-min exposure to the analyte and another 20-min without any exposure. The volume of analytes on the gas chamber was 10 mL with a minimum incubation period of 10 min before the test.

## 3 Results and discussion

### 3.1 Polyaniline synthesis

**Fig. 4:**
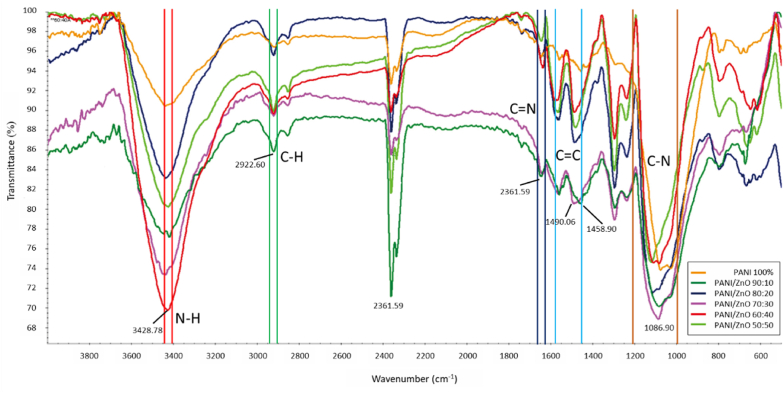
Ftir of all samples.

The most conductive form of PANI is polymeraldine salt. It has a dark green color at the end of the polymerization process. The color of polymeraldine salt can be identified visually. All samples with ZnO loading shows the same color as the polyaniline without ZnO loading. The present study used FTIR was carried out to analyze the surface functional groups of the polyaniline powder. Several noticeable IR peaks for PANI are quinonoid and benzenoid ring vibration [43,44]. The general structure of PANI contains *N*–H, *C*–N, *C*–H, C=N, and C=C aromatic ring bonding. The summary of important peaks is detailed in Appendix A2, and FTIR graphs are presented in Fig. 4. The graph confirms the general structure of polyaniline within all sample-types.

### 3.2 Surface morphology and elemental composition

**Fig. 5:**
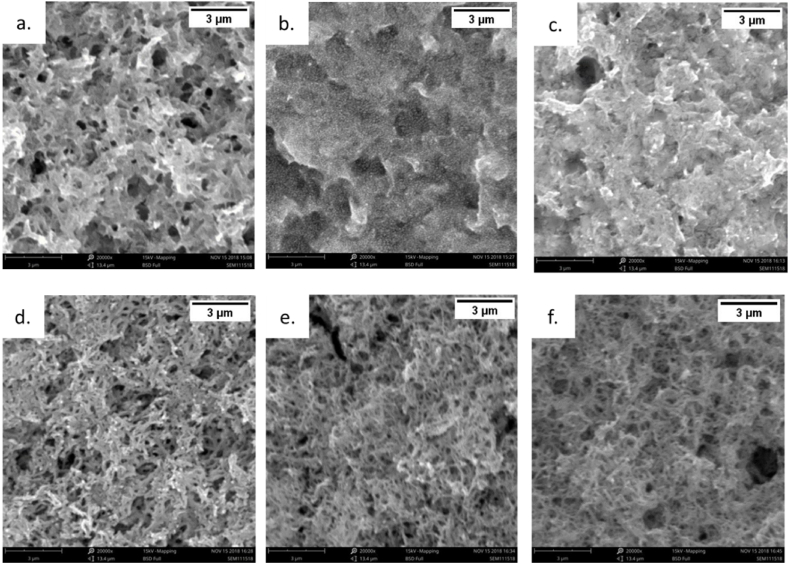
SEM Images of a) PANI 100%; b) PANI/ZnO 90:10; c) PANI/ZnO 80:20 d) PANI/ZnO 70:30; e) PANI/ZnO 60:40; f) PANI/ZnO 50:50.

The surface morphology of a gas sensor plays an essential role since the interaction between the analytes and the sensor depends on the active surface of the sensing materials i.e., surface area. All scanning electron microscopy images are presented in Fig. 5(a–f) in 20 k x magnification.

Visible pores on the surface of the pure-PANI sample (see Fig. 5(a)) can be observed. The porousness may make the sensing surface susceptible to changes in absorption and consequently the overall performance. High density pores are occurred in Fig. 5 (a, d,e, and f) or PANI/ZnO ratio from 100%, 70%, 60%, and 50%, where lesser pores occurred in Fig. 5(b–c) or (90% and 80%). A greater number of pores in the surface indicates these surfaces are rough and uneven. The PANI/ZnO 70:30 samples can be seen in Fig. 5(d). A network of fibrous structures is formed by PANI polymerization below its surface. Sprouted thread-like structures have formed towards the surface for PANI/ZnO 60:40 and 50:50 as shown in Fig. 5(e–f). These structures have a mesh-like an appearance on the top of the surface, which subsequently increases the sample's surface area-to-volume ratio to the analyte. From this phenomenon, it can be inferred that lowering PANI wt.% can increase the surface area due to favorable polymerization and net-like structural formation, thereby promoting the surface area-to-volume ratio.

**Fig. 6:**
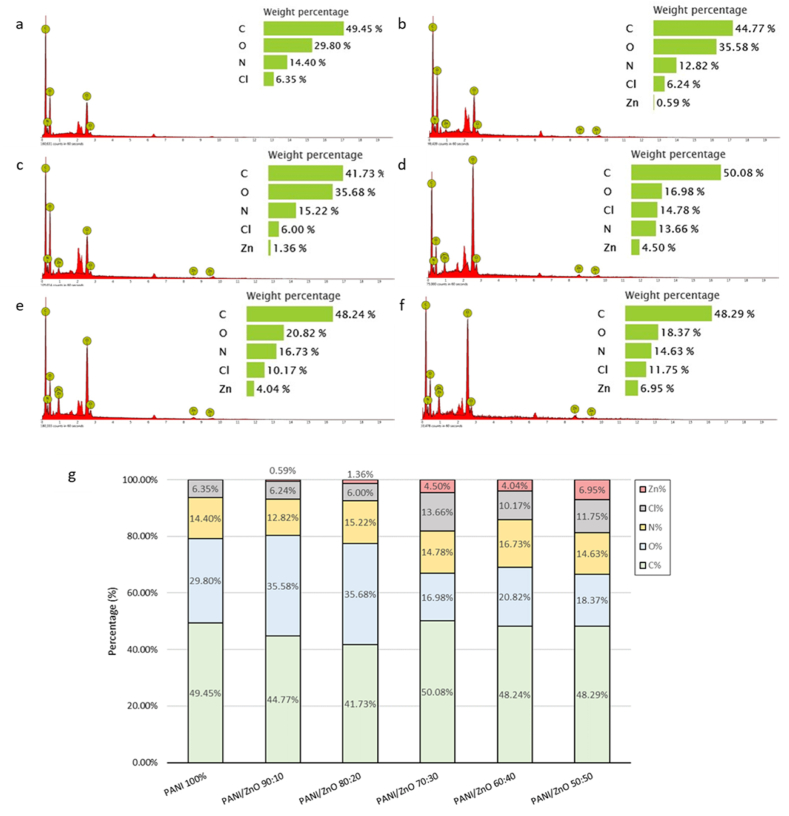
EDX Images of a) PANI 100%; b) PANI/ZnO 90:10; c) PANI/ZnO 80:20 d) PANI/ZnO 70:30; e) PANI/ZnO 60:40; f) PANI/ZnO 50:50 g) Compilation of elemental compositions of all samples.

Elemental analysis using EDX was conducted to confirm the incorporation of ZnO into PANI. The element of pure-PANI almost has the same composition as All PANI/ZnO composites. Elemental compositions are presented in Fig. 6(a–g). Based on the result, the zinc (Zn) element is present in all PANI/ZnO composites samples. The result confirmed that the incorporation of zinc oxide (ZnO) into the PANI/ZnO composite was successful. Nitrogen (N) and carbon (C) are also present and are in alignment with the results obtained via FTIR analysis and the chemical structure of PANI. Other elements like chlorine and oxygen are present due to the acidic medium used and the oxidative polymerization process during the synthesis process. The results from EDX output also revealed that the content of ZnO slightly decreased in the ratio 60:40 and the N element increased. These results were an indication that the effect of mixing where the inhomogeneous dispersion of the element can give a linear effect to the sensing performance.

### 3.3 Resistance measurement

**Fig. 7:**
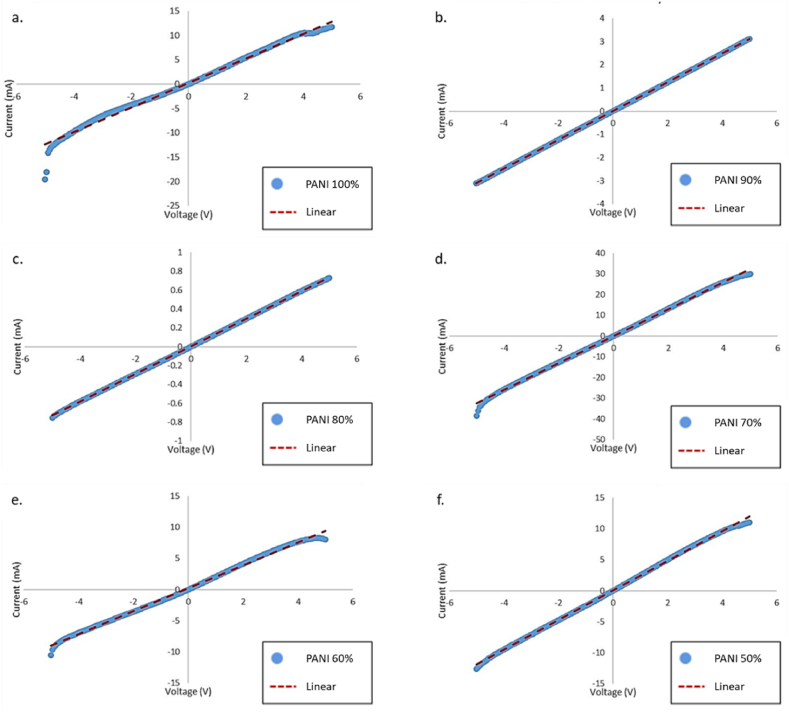
I–V curve profile of a) PANI 100%; b) PANI/ZnO 90:10; c) PANI/ZnO 80:20; d) PANI/ZnO 70:30; e) PANI/ZnO 60:40; f) PANI/ZnO 50:50.

I–V characterization of PANI and PANI/ZnO was performed to obtain the behavior and durability of the sensor. Resistance measurements of all samples are presented in Appendix A3. Based on the slope of I–V curve graphs in Fig. 7, Impedance values are greater than resistance values. The frequency from the AC signal causes additional drift in the increasing resistance. The alternating current (AC) current creates a changing magnetic field that makes an electric field at the center of the sample as a result of the skin effect. This phenomenon pushes electrons to the sides of the sample and reduces the current density at the center, hence increasing the resistance. The skin effect does not occur in direct current (DC) resistance because of the different resistance between DC and AC. Therefore, the coefficient of determination (R^2^) for all samples is equivalent to 1. This phenomenon can be seen in Fig. 7(a–f), all trendlines close to all corresponding data points.

**Fig. 8:**
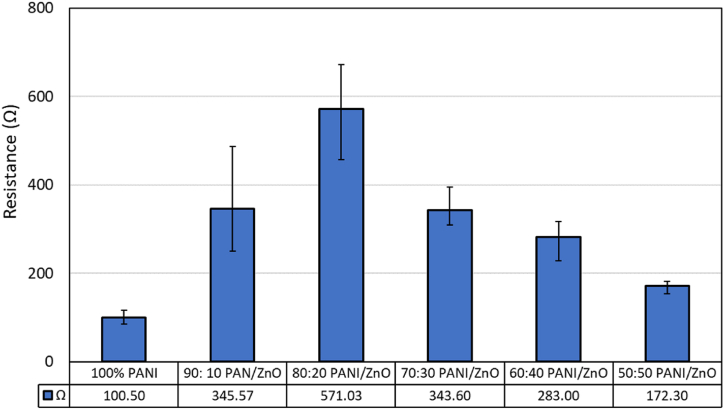
Average resistance of PANI and PANI/ZnO composites.

From Fig. 7, the input bias of all samples is -5 V–5 V. All the samples exhibit Ohmic behavior at this range. However, the resistance measurement cannot be performed. The results showed bias when the input is changed from −10 V to 10 V. This behavior confirms that the sensors were tested at their limit, i.e., breakdown voltage range. Therefore, the working range of the sensor when connected to a circuit is -5 V–5 V. From Fig. 7(a–f), the graphs also resemble the I–V curve graphs of the resistor. The graphs of pure- PANI, PANI/ZnO 70:30, and 60: 40 show a non-linear response while PANI/ZnO 90:10 and 80:20 show a linear response. Furthermore, gas sensing measurements were performed for 15 samples for every sample-type and the detailed results can be seen in Appendix A4. The average resistance is shown in Fig. 8. All sample variations have a large spread error bar representing standard deviation. The ratio between solvent and binder shows variations in the current state and requires a separate study to increase reproducibility.

### 3.4 Gas sensing analysis

#### 3.4.1 Ammonia

**Fig. 9:**
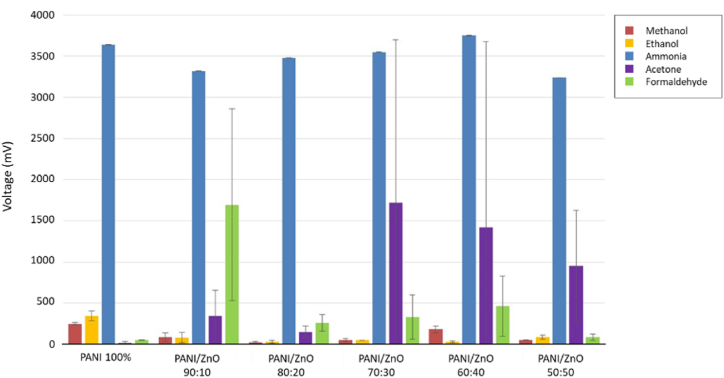
Average response of PANI and PANI/ZnO sensors to all analytes.

Based on the results obtained, ammonia shows an incredible response when compared to other analytes evaluated (see Fig. 9). The sensor works with the analyte exposure time for 1200 s and the unexposed analyte time for 1200 s. One time of exposure and unexposed of the gas makes one complete cycle. Thus, 0–2400s is the first cycle, 2400 s–4800 s is the second cycle, and 4800–7200s is the third cycle.

**Fig. 10:**
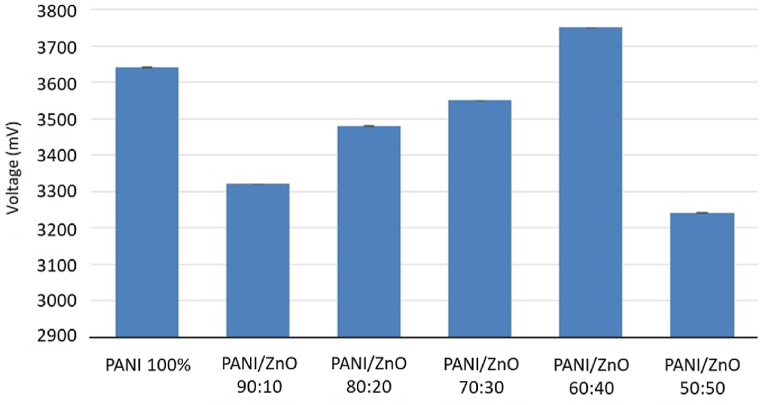
Response of ammonia in all sensors.

A radar plot is used to present the selectivity of the gas sensor toward the analyte, ammonia. The response value of each variation is illustrated in Fig. 10. The detailed samples exposure results are presented in Appendix A5. Moreover, it is shown that the response times for the PANI/ZnO with the ratio of PANI/ZnO 80:20 and 70:30 are more significant, which are beyond the exposure time. The results from Fig. 10 also revealed that sensing in the ratio 60:40 has the highest voltage compared with other ratios.

#### 3.4.2 Acetone and formaldehyde

**Fig. 11:**
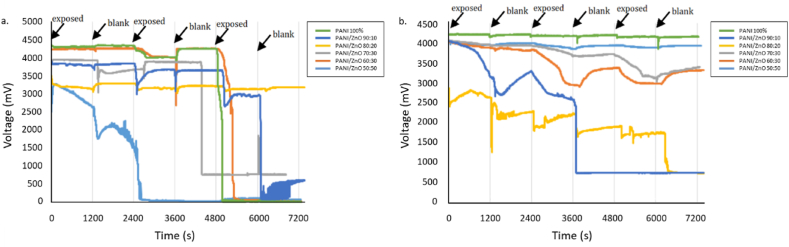
Overlay response graphs of all sensors on a) Acetone; b) Formaldehyde.

PANI and PANI/ZnO sensors did not demonstrate strong responsivity to acetone and formaldehyde gases (see Fig. 9). Based on the results from the sensing experiments, the sensors do not show repeatability upon exposure to acetone and formaldehyde. Overlay graphs of the sensor on these gases’ exposure is shown in Fig. 11(a–b).

From Fig. 11(a), all sensors have damage after acetone exposure except PANI/ZnO 80:20. Then, formaldehyde exposure damaged PANI/ZnO 90:10 and PANI/ZnO 80:20 and poisoned PANI/ZnO 70:30 and PANI/ZnO 60:40 (see Fig. 11(b)). PANI/ZnO 70:30 and 60:40 sensors did not respond within the recovery time of the first and second cycle as indicated by ‘zero’ value (see Appendix A5). Zero value implies the voltage change was recorded to be negligible. Consequently, the time was not recorded either. PANI/ZnO 90:10 and 80:20 sensors have incurred damage respectively before and during the start of the third cycle (see Appendix A5), as indicated by a sudden voltage drop in Fig. 11(b). Furthermore, the addition of ZnO negatively impacts this sensor. The sensors demonstrate little to no-damage only in samples with the highest ZnO loading (PANI/ZnO 50:50).

#### 3.4.3 Methanol and ethanol gas sensing

**Fig. 12:**
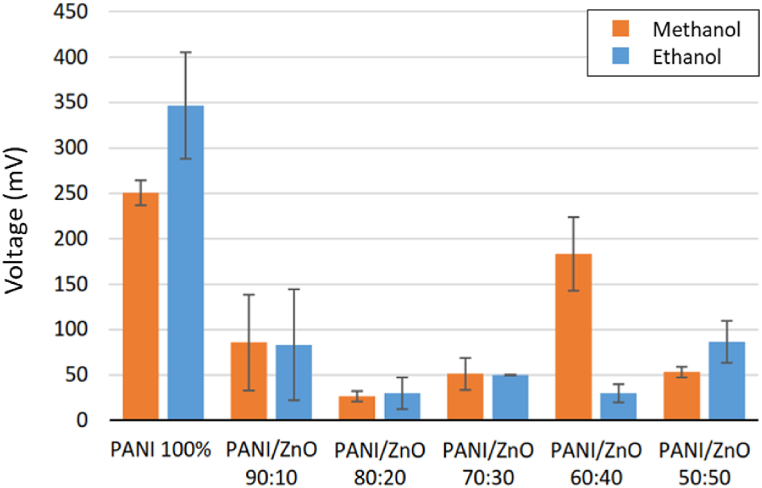
Sensor response of all sensors on both methanol and ethanol.

PANI and PANI/ZnO sensors show an excellent response to methanol and ethanol exposure (see Fig. 12). Pure-PANI has the best response to methanol and ethanol exposure than the composite sensors. However, repeatability could not be achieved as the pure-PANI sensors were damaged during the third cycle and showed an inconsistent signal result. This response confirms the hypothesis about the susceptibility of pure-PANI being poisoned by methanol and ethanol exposure, rendering it inadept for further cycles of measurement.

All PANI/ZnO sensors were not damaged but slightly poisoned. Sensors with lowered degrees of poisoning were shown to be loaded with ZnO, confirming that PANI/ZnO in the acceptable ratio has a positive influence of the stability of the sensors preventing it from poisoning induced damage. All sensors have less than 100 s response time in the first cycle of methanol exposure (see Appendix A5). This trend is also observed upon ethanol exposure except on PANI/ZnO 80:20 and 60:40. Lastly, PANI/ZnO 90:10, 80:20, and 70:30 have identical sensor responses towards exposure of alcohol analytes.

### 3.5 Selectivity

**Fig. 13:**
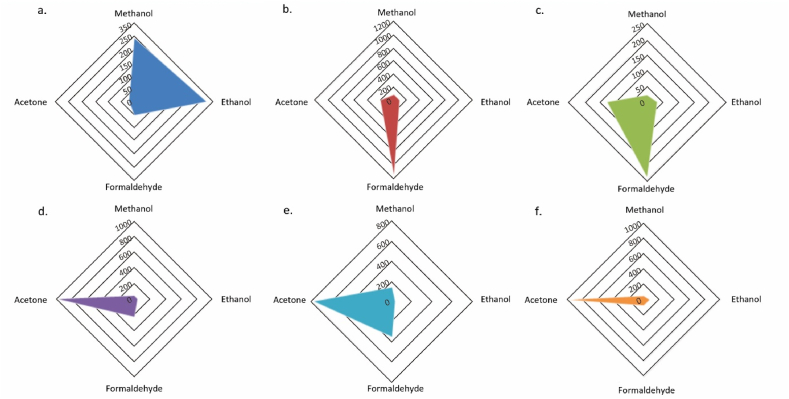
Pattern recognition of all sensors to analytes a) PANI 100%; b) PANI/ZnO 90:10; c) PANI/ZnO 80:20; d) PANI/ZnO 70:30; e) PANI/ZnO 60:40; f) PANI/ZnO 50:50.

Pattern recognition is used to identify analyte selectivity when tested on PANI and PANI/ZnO sensors. Fig. 13(a–f) shows the sensor selectivity to four analytes – acetone, formaldehyde, methanol, and ethanol. The unique collective sensor response on different analytes makes it easily distinguishable from one-another. Fig. 13 (a) shows a good selectivity of pure-PANI to methanol and ethanol. PANI/ZnO 90:10 is selective to formaldehyde (see Fig. 13 (b)). The selectivity of PANI/ZnO 50:50 to acetone is shown in Fig. 13 (f). From Fig. 13(c–e), PANI/ZnO 80:20, 70:30, and 60:40 sensors are selective to acetone and formaldehyde.

**Fig. 14:**
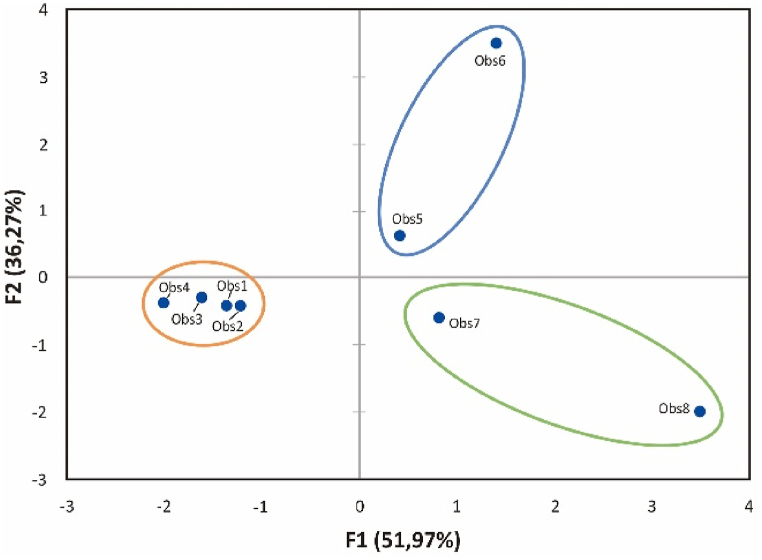
PCA plot of the sensors when exposed to acetone, formaldehyde, methanol, and ethanol (Observation axes F1 and F2 88.24%).

Principal component analysis was done to establish and distinguish between the sensor's response to different analyte exposure, namely acetone, formaldehyde, methanol, and ethanol, as shown in Fig. 14. Two trials were performed and displayed per analyte. Observation 1–4 represents methanol and ethanol, 5–6 for acetone, and 7–8 for formaldehyde (Appendix A6). Three groups formed, and sensor responses on methanol and ethanol were clustered. The difference in sensor responses corresponds to the classification based on their chemical structure, i.e., acetone (ketone), formaldehyde (aldehyde), and methanol-ethanol (alcohol). Thereby, extending the sensing capabilities to detect/respond to specific class of functional groups such as ketone, aldehyde, and alcohol.

### 3.6 Calibration curve of PANI and PANI/ZnO sensors

**Fig. 15:**
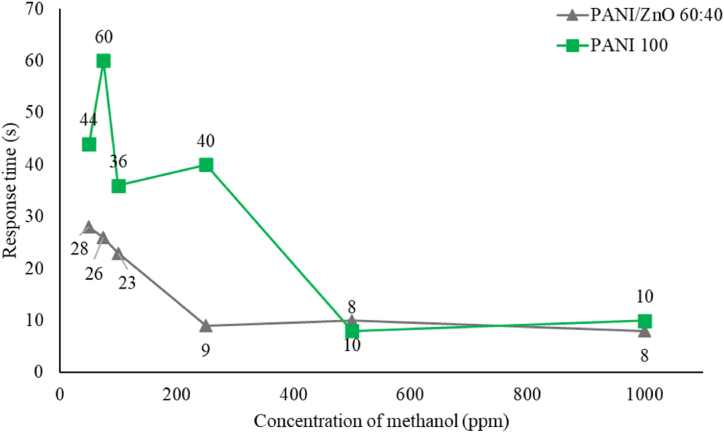
Response time vs. concentration graph of PANI/ZnO 60:40 and PANI 100%.

The calibration curve was used to identify the relationship between sensor response or change in voltage and the analyte concentration. The detailed calibration processes are described in Appendix A7. The study showed that the relationship between methanol concentration and sensor response time can be seen in Fig. 15. For PANI/ZnO 60:40, below 200 ppm of methanol concentration results in a higher response time value (23–28 s). It means the sensor takes longer to respond to methanol molecules. The sensor demonstrates a quicker response time for 200–1000 ppm of methanol concentration (8–10 s). Pure-PANI has a longer response time below 500 ppm of methanol concentration (40–50 s). Higher concentration of methanol (500–1000 ppm), the sensor demonstrates a quicker response time (8–10 s). Pure-PANI sensor needs a longer response time for smaller methanol concentrations as opposed to PANI/ZnO 60:40.

## 4 Future trends

The superior properties of PANI make this conductive polymer ubiquitous in the field of electronic-applications. The doping method to form PANI nanocomposite to improve the transport mechanism using different materials such as metal oxides and graphene has a prospect in the structure and chemical applications. Moreover, the PANI-RGO-ZnO nanocomposite shows excellent electrochemical activity, great capacitance, and fast charge-discharge that can facilitate the demand for energy storage and battery applications in the future [45,46]. The need for clean energy has recently increased, with a special focus on photovoltaics. The material selection of solar cells is one of the challenges for its application. High-cost and low-efficiency problems require continuous research and development. As an intrinsically conductive polymer, PANI has greater prospects for application as cell material. PANI can provide better efficiency, low cost, and ultra-thin solar cells. A combination of different materials of PANI/ZnO also can enhance solar cell performance [47]. In the future, PANI can be extensively used in coatings for corrosion protection with the addition of epoxy and ZnO. The substantial performance is obtained as a result of incorporating PANI/ZnO composite into the epoxy coating. Other parallel fabrication routes, such as incorporating core-shell nanoparticles into Epoxy/PANI/ZnO composite, can further enhance the self-healing behavior of base metals such as anodized aluminum [48,49].

## 5 Conclusion

PANI and PANI/ZnO sensors were fabricated successfully in this work. Fourier Transform Infrared Spectroscopy (FTIR) and Energy Dispersive X-ray Spectroscopy (EDX) were used to identify the sensing materials' functional groups and elemental compositions. Pores were inevitably formed on the surface of the samples resulting in rough and uneven surfaces. The high density of pores mostly occurred in the PANI/ZnO ratio of 100%, 70%, 60%, and 50%. These high pores can be indication that the sensor have less resistances. Fibrous structures were formed in the following ratios: 70:30, 60:40, and 50:50 for the PANI/ZnO composites. Pure-PANI has the lowest average resistance with 100.5 Ω. The highest resistance achieved was 571.03 Ω by PANI/ZnO 80:20. This behavior indicated that the incorporation of ZnO in the PANI matrix modified its resistance. The resistance of PANI/ZnO showed that less pores exhibited higher resistance as shown in combination PANI/ZnO 90:10 and 80:20. The variability of resistances confirms the inhomogeneous dispersion of ZnO within the PANI matrix. Moreover, pure-PANI and PANI/ZnO sensors were selective to ammonia exposure with an average sensor response of 3496.67 mV and invariably succumb to poisoning and incur damage rendering them defunct for further experimental rounds. The addition of ZnO in the PANI matrix increased its stability towards methanol exposure. A radar plot was used to identify the unique sensor response patterns towards ammonia, acetone, formaldehyde, methanol, and ethanol. Pattern recognition was formed from four analytes to identify the sensor selectivity of acetone, formaldehyde, methanol, and ethanol. Principal component analysis (PCA) identified ketone, aldehyde, and alcohol groups. Therefore, the sensors were able to distinguish all the analytes respectively. The mixing method between solvent and binder ratio will be optimized in further research in the future in order to maximize reproducible responsivity of these sensors.

## Author contribution statement

Juanito Raphael F. Foronda: Conceived and designed the experiments; Performed the experiments; Analyzed and interpreted the data; Contributed reagents, materials, analysis tools or data; Wrote the paper.

Lugas Gada Aryaswara: Performed the experiments; Analyzed and interpreted the data; Contributed reagents, materials, analysis tools or data; Wrote the paper.

Gil Nonato C. Santos: Conceived and designed the experiments; Contributed reagents, materials, analysis tools or data; Wrote the paper.

Swathi N·V Raghu: Analyzed and interpreted the data; Wrote the paper.

Muhammad Akhsin Muflikhun: Conceived and designed the experiments; Contributed reagents, materials, analysis tools or data; Wrote the paper.

## Funding statement

This research did not receive any specific grant from funding agencies in the public, commercial, or not-for-profit sectors.

## Data availability statement

Data will be made available on request.

## Declaration of interest's statement

The authors declare that they have no known competing financial interests or personal relationships that could have appeared to influence the work reported in this paper.
