# Supplementary material for: Broad-class volatile organic compounds (VOCs) detection via polyaniline/zinc oxide (PANI/ZnO) composite materials as gas sensor application
Source: Heliyon. 2023 Feb 5;9(2):e13544. doi: 10.1016/j.heliyon.2023.e13544 (PMC9929445; doi:10.1016/j.heliyon.2023.e13544)
Supplement: Multimedia component 1 [file mmc1.docx]

Broad-class Volatile organic compounds (VOCs) detection via Polyaniline/Zinc oxide (PANI/ZnO) composite materials as gas sensor application

Appendix

A1. Detailed methods

Six different weight percentage ratios (% wt.) were evaluated in this research and the values of aniline and zinc oxide are represented in Table A.1. The solution was prepared from 100 mL of 1 M HCl mixed with aniline and zinc oxide measurement. Ammonium persulfate solution was prepared from 1.141 g ammonium persulfate mixed with 100 mL of 1 M HCl. Ammonium persulfate was added dropwise into each ratio-variation of aniline solutions and stirred for an hour. These solutions were tagged as polyaniline solutions. After that, the polyaniline solutions were cooled to lengthen their polymerization. All solutions were subjected to sonication for 10 minutes prior to the filtration process. During the filtration process, each ratio-variation of the polyaniline solutions was filtered by Whatman 589/3 ash less filter, assisted by vacuum. Post filtration each ratio-variation sludge was divided into two batches. The first batch was oven-dried at 90^o^C for an hour, then crushed using mortar-pestle to pulverize into powder and the second batch was subjected to mixing using a vortex mixer with tetrahydrofuran and laminating fluid that acted as solvent and binder respectively. In each ratio-variation, 0.11 g of PANI or PANI/ZnO, 0.5 mL tetrahydrofuran, and 0.1 laminating fluid were used as the working ratio of solutions for the sensors.

Table A.1. Measurement of aniline and zinc oxide with their respective PANI/ZnO ratio

| Ratio | Aniline (mL) | Aniline (g) | Zinc Oxide (g) |
| --- | --- | --- | --- |
| PANI 100% | 0.91 mL | 0.93 g | 0 g |
| PANI/ZnO 90:10 | 0.82 mL | 0.837 g | 0.093 g |
| PANI/ZnO 80:20 | 0.73 mL | 0.744 g | 0.186 g |
| PANI/ZnO 70:30 | 0.64 mL | 0.651 g | 0.279 g |
| PANI/ZnO 60:40 | 0.55 mL | 0.558 g | 0.372 g |
| PANI/ZnO 50:50 | 0.45 mL | 0.465 g | 0.465 g |

A2. FTIR peaks results

FTIR spectral showed that there are several important peaks discovered after the tests. The summary of important peaks is shown in Table A.2. Results confirm the general structures of polyaniline. The general structures of polyaniline contain N-H, C-H, C=N, C=C, and C-N.

Table A.2. Summary of important peaks on IR spectra of all samples (cm^-1^)

|  | PANI | PANI/ZnO | | | | |
| --- | --- | --- | --- | --- | --- | --- |
|  | 100% | 90:10 | 80:20 | 70:30 | 60:40 | 50:50 |
| N - H | 3438.64 | 3420.98 | 3439.31 | 3438.66 | 3428.78 | 3425.57 |
| C - H | 2920.79 | 2922.60 | 2924.01 | 2923.31 | 2923.65 | 2924.34 |
| C = N | 1645.48 | 1645.62 | 1645.52 | 1663.68 | 1638.26 | 1645.69 |
| C = C | 1456.45 | 1466.90 | 1565.09;  1488.54 | 1563.08;  1490.06 | 1578.27;  1488.88 | 1588.23;  1491.86 |
| C - N | 1076.73 | 1088.90 | 1237.64;  1112.43 | 1088.40 | 1238.58;  1028.88 | 1240.99;  1120.99 |

A3. Resistance results

Resistance measurement was performed using a simple fluke multimeter. The durability and the behavior of the sensors were obtained from I-V characterization. All samples have the same value equivalent to 1 for the coefficient of determination (R^2^). The impedance value is greater than resistance because of skin effect. Table A.3 contains the summary of resistance measurement.

Table A.3.1 Resistance measurement of all samples

|  | Resistance  from multimeter | Slope from  I-V curve (mA vs V) | Impedance measurement (1 / slope) | R^2^ |
| --- | --- | --- | --- | --- |
| PANI 100% | 100.5 Ω | 1.48 | 674.3088 Ω | 1 |
| PANI/ZnO 90:10 | 345.57 Ω | 0.62 | 1610.05 Ω | 1 |
| PANI/ZnO 80:20 | 571.03 Ω | 0.15 | 6825.94 Ω | 0.99 |
| PANI/ZnO 70:30 | 343.6 Ω | 6.48 | 154.4 Ω | 0.99 |
| PANI/ZnO 60:40 | 283 Ω | 1.84 | 544.48 Ω | 0.99 |
| PANI/ZnO 50:50 | 172.3 Ω | 2.38 | 419.66 Ω | 0.99 |

Table A3.2 Total resistance data measurement

| Sample No. | 100% PANI | 90: 10 PAN/ZnO | 80:20 PANI/ZnO | 70:30 PANI/ZnO | 60:40 PANI/ZnO | 50:50 PANI/ZnO |
| --- | --- | --- | --- | --- | --- | --- |
| Sample 1 | 86.00 | 487.20 | 672.00 | 394.60 | 317.00 | 154.00 |
| Sample 2 | 117.20 | 299.50 | 584.00 | 326.50 | 303.00 | 181.90 |
| Sample 3 | 98.30 | 250.00 | 457.10 | 309.70 | 229.00 | 181.00 |
| Average | 100.50 | 345.57 | 571.03 | 343.60 | 283.00 | 172.30 |
| max | 16.70 | 141.63 | 100.97 | 51.00 | 34.00 | 9.60 |
| min | 14.50 | 95.57 | 113.93 | 33.90 | 54.00 | 18.30 |

A4. Statistical results

The results from the measurement of all samples showed that ZnO loading into the PANI matrix increases the mean resistance value between 30 ~ 10^3^ Ω (see Table A.4). Based on the result, the resistance range of less than or equal to 10^3^ Ω has a good sensing response. This study focuses on the development and application of PANI and ZnO composites to be applied as potential gas sensing devices. The optimization between solvent and binder ratio was not performed within these set of experiments. It is pertinent to note that, there is no strong relationship between the ZnO ratio on the resistance values obtained within these experiments. Furthermore, the dispersion of ZnO in the PANI matrix is not entirely homogeneous.

Table A.4. Mean, standard deviation, and coefficient of variation values of all samples

|  | 100% PANI | 90:10 PANI/ZnO | 80:20 PANI/ZnO | 70:30 PANI/ZnO | 60:40 PANI/ZnO | 50:50 PANI/ZnO |
| --- | --- | --- | --- | --- | --- | --- |
| Mean (Ω) | 80.37 | 303.77 | 202.27 | 279.79 | 196.41 | 132.07 |
| Deviation (Ω) | 46.75 | 265.68 | 225.64 | 380.57 | 177.43 | 66.69 |
| Coefficient of Variance | 0.98 | 0.87 | 1.11 | 1.36 | 0.9 | 0.5 |

A5. Sensor performance

From Table A.5, all samples exposed to ammonia were only performed in one cycle and the subsequent recovery times were not recorded due to sensor damage. All samples exposed to acetone have damage except for PANI/ZnO 80:20. Formaldehyde exposure damaged 90:10 and 80:20 PANI/ZnO and Poisoned PANI/ZnO 70:30 and 60:40. All samples exposed to methanol and ethanol were not damaged.

Table A.5. Response time and recovery time of all sensors on all analytes

| Analytes | Ratio Variations | Response Time | | | Recovery Time | | |
| --- | --- | --- | --- | --- | --- | --- | --- |
|  |  | Cycle 1 | Cycle 2 | Cycle 3 | Cycle 1 | Cycle 2 | Cycle 3 |
| Ammonia | PANI 100% | 306.4 – 400.2 | Break | | | | |
|  | PANI/ZnO 90:10 | 41.6 – 400 |  |  |  |  |  |
|  | PANI/ZnO 80:20 | 1332.4 – 1530.2 |  |  |  |  |  |
|  | PANI/ZnO 70:30 | 1214.4 – 1303.2 |  |  |  |  |  |
|  | PANI/ZnO 60:40 | 100 – 1500 |  |  |  |  |  |
|  | PANI/ZnO 50:50 | 50.2 – 500 |  |  |  |  |  |
| Acetone | PANI 100% | 100 - 202.9 | 33.2 - 500.3 | 22.2 - Break | 100.2 - 300 | 55 - 304.2 | Break |
|  | PANI/ZnO 90:10 | 10.8 - 702.8 | 34.1 - 800 | 185.8 - 732 | 200 - 900 | 0 | Break |
|  | PANI/ZnO 80:20 | 6.7 - 600.1 | 70 - 400 | 209 - 600 | 32.4 - 400 | 121.5 - 600 | 400 - 900 |
|  | PANI/ZnO 70:30 | 100 - 1123 | 0 | Break | 500 - 1400 | Break | Break |
|  | PANI/ZnO 60:40 | 0 | 200 - 700 | 105 - Break | 100 - 202.8 | 43.2 - 302 | Break |
|  | PANI/ZnO 50:50 | 200.7 - 1200 | Break | Break | 202.6 - 600.8 | Break | Break |
| Formaldehyde | PANI 100% | 1125.3 - 1200 | 14 - 61 | 75.2 - 210 | 100 - 800 | 400 - 900 | 0 |
|  | PANI/ZnO 90:10 | 500 - 1200 | 33 - 800 | Break | 100 - 1200 | Break | Break |
|  | PANI/ZnO 80:20 | 16.7 - 816.5 | 59 - 300 | 305.3 - 420 | 401 - 1200 | 200.9 - 1200 | Break |
|  | PANI/ZnO 70:30 | 402. - 1200 | 113 - 1200 | 114 - 1021 | 0 | 0 | 119.4 - 1200 |
|  | PANI/ZnO 60:40 | 300 - 1200 | 207 - 1100 | 173 - 917 | 0 | 240 - 1000 | 203.3 - 1100 |
|  | PANI/ZnO 50:50 | 100 - 1000 | 200 - 1200 | 278 - 900 | 227 - 600 | 110.3 - 1000 | 166 - 737.2 |
| Methanol | PANI 100% | 40 - 580 | 83 - 380 | Break | 290 - 380 | 288 - 400 | Break |
|  | PANI/ZnO 90:10 | 9 - 350 | 250 - 350 | 150 - 350 | 50 - 950 | 60 - 970 | 156 - 350 |
|  | PANI/ZnO 80:20 | 68 - 223 | 567 - 1133 | 103.2-557 | 0 | 467 - 600 | 0 |
|  | PANI/ZnO 70:30 | 35 - 571 | 370 - 883 | 270 - 670 | 670 - 900 | 400 - 1000 | 521 - 1100 |
|  | PANI/ZnO 60:40 | 6 - 160 | 135 - 360 | 141 - 360 | 146 - 460 | 104 - 660 | 80 - 460 |
|  | PANI/ZnO 50:50 | 32 - 180 | 118 - 670 | 70 - 161 | 61 - 261 | 64 - 361 | 161 - 661 |
| Ethanol | PANI 100% | 30 - 1100 | 13 - 1000 | 7 - 1000 | 20 - 280 | 20 - 200 | 20 - 500 |
|  | PANI/ZnO 90:10 | 45 - 545 | 258 - 845.6 | 193.7 - 545 | 145 - 1045 | 167.3 - 1145 | 245 - 945 |
|  | PANI/ZnO 80:20 | 127.7 - 500 | 400 - 1000 | 574.7 - 900 | 400 - 1001 | 0 | 0 |
|  | PANI/ZnO 70:30 | 11.1 - 356.3 | 307.1 - 500 | 953.1 - 1100 | 145.7 - 309.5 | 776.7 - 1100 | 0 |
|  | PANI/ZnO 60:40 | 200 - 300 | 400 - 600.2 | 400 - 600 | 800 - 1000 | 800 - 1100 | 400 - 800 |
|  | PANI/ZnO 50:50 | 25 - 400.7 | 120.9 - 3300 | 69.2 - 300 | 152.1 - 605.6 | 138.6 - 300 | 85.9 - 501.4 |

A6. Selectivity analysis

As discussed earlier in the article, the highest sensor response was recorded upon exposure to ammonia as opposed to (acetone, formaldehyde, methanol, ethanol) as shown in Fig. A6. However, ammonia exposure results in permanent damage to the sensing surface-material. This reason is used to rule out ammonia from this comparison to the other analytes. After ruling out ammonia from the selectivity comparison due to lack of sensor repeatability post gas-exposure, pattern recognition is formed from four kinds of analytes.


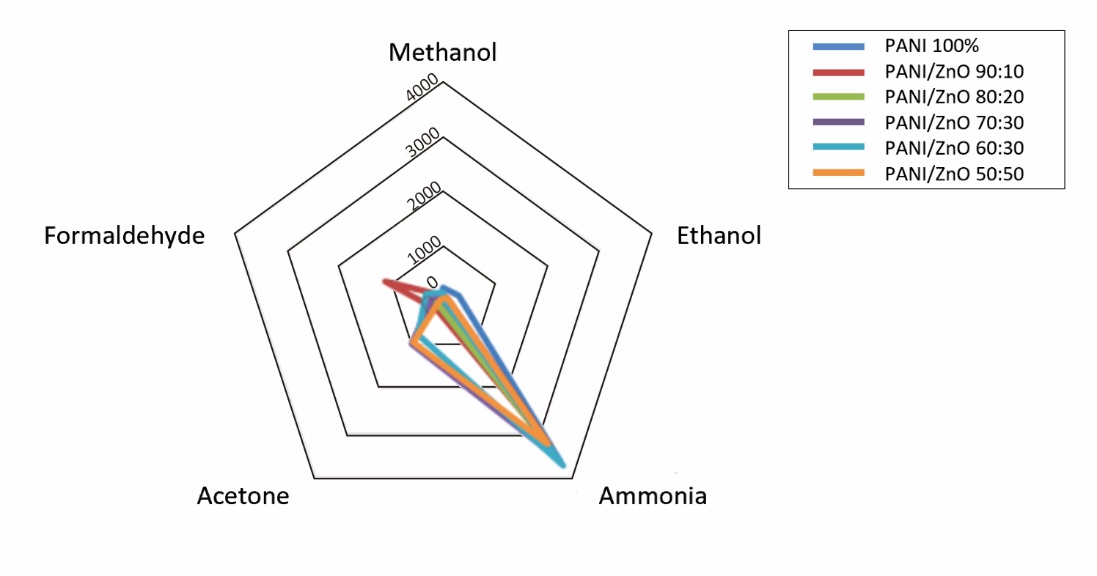


Fig. A6. Radar plot overlap of all sensors on all gasses

Table A.6. Principal component analysis of the sensor

|  |  | PANI  100% | PANI/ZnO  90:10 | PANI/ZnO  80:20 | PANI/ZnO  70:30 | PANI/ZnO  60:40 | PANI/ZnO  50:50 |
| --- | --- | --- | --- | --- | --- | --- | --- |
| 1 | Methanol | 260.27 | 137.59 | 30 | 43.4 | 160 | 50 |
| 2 | Methanol | 240.8 | 32.1 | 30 | 39.24 | 230 | 60 |
| 3 | Ethanol | 280 | 30 | 50 | 50 | 40 | 60 |
| 4 | Ethanol | 370 | 150 | 20 | 50 | 20 | 100 |
| 5 | Acetone | 10 | 110 | 230 | 320 | 30 | 470 |
| 6 | Acetone | 30 | 230 | 140 | 3120 | 220 | 1430 |
| 7 | Formaldehyde | 60 | 870 | 270 | 110 | 140 | 80 |
| 8 | Formaldehyde | 50 | 2520 | 360 | 260 | 860 | 130 |

A7. Calibration

Methanol was used as the analytes and pure-PANI and 60:40 PANI/ZnO as sensors. Those sensors were chosen because of their excellent selectivity to methanol. Fig. A7.1(a-b) shows calibration curve graphs of 60:40 PANI/ZnO and pure- PANI sensors. PANI/ZnO 60:40 and PANI 100% have almost the same value at 500 ppm and 1000 ppm.

A linear relationship between change in voltage and concentration can be determined after changing the x-axis into a logarithmic scale, as shown in Fig. A7.2(a-b). PANI/ZnO 60:40 has a slope of 132.36, and pure-PANI has a slope of 271.13. The highest slope is gained by pure-PANI, which implies an enormous change in voltage per concentration. The coefficient of determination (R^2^) for PANI/ZnO 60:40 and pure-PANI are 0.9655 and 0.9344. Experimental data points for PANI/ZnO 60:40 and pure-PANI show close to their regression line. PNI/ZnO 60:40 has the larger value of R^2^, demonstrating a nearly perfect overlay of experimental findings to the fit- regression line. Based on these graphs, we infer that the addition of ZnO to the PANI sensor will increase its stability whilst negatively impacting its responsivity, up to a certain concentration.


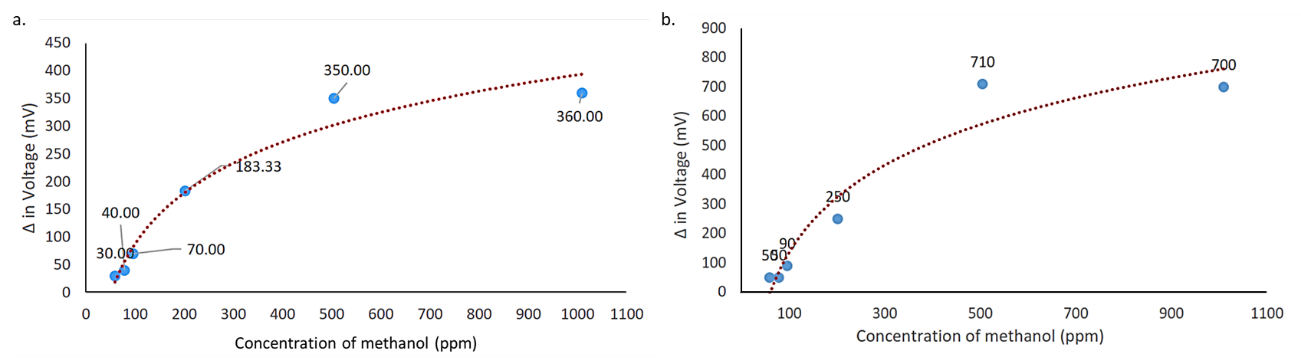


Fig. A7.1 Calibration curve of a) PANI/ZnO 60:40; b) PANI 100%


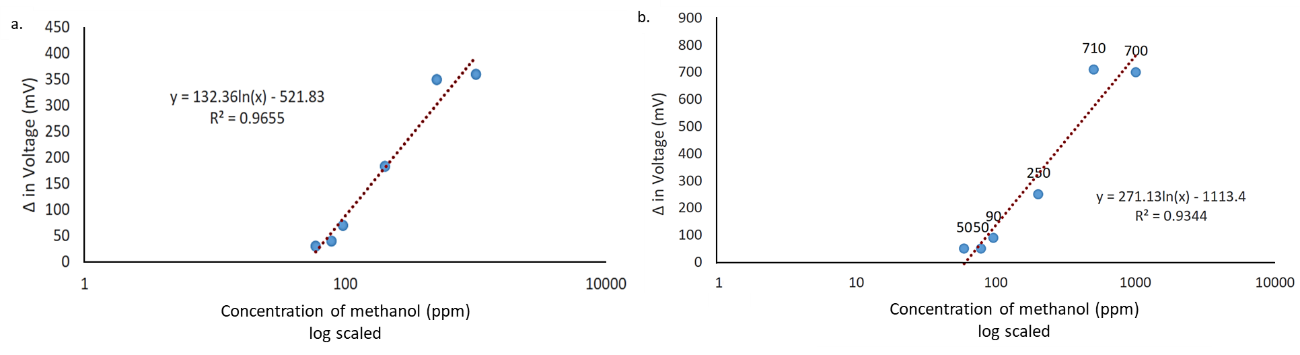


Fig. A7.2 Logarithmic scaled calibration graph of a) PANI/ZnO 60:40; b) PANI 100%
